# Molecular Signals Controlling the Inhibition of Nodulation by Nitrate in *Medicago truncatula*

**DOI:** 10.3390/ijms17071060

**Published:** 2016-07-02

**Authors:** Giel E. van Noorden, Rob Verbeek, Quy Dung Dinh, Jian Jin, Alexandra Green, Jason Liang Pin Ng, Ulrike Mathesius

**Affiliations:** Division of Plant Science, Research School of Biology, Australian National University, Canberra ACT 2601, Australia; giel.vannoorden@anu.edu.au (G.E.v.N.); verbeekr@hotmail.com (R.V.); quydung.dinh@gmail.com (Q.D.D.); jinjian29@hotmail.com (J.J.); u5013038@anu.edu.au (A.G.); Jason.Ng@anu.edu.au (J.L.P.N.)

**Keywords:** auxin, flavonoid, nitrate, nodulation, proteomics, reactive oxygen species

## Abstract

The presence of nitrogen inhibits legume nodule formation, but the mechanism of this inhibition is poorly understood. We found that 2.5 mM nitrate and above significantly inhibited nodule initiation but not root hair curling in *Medicago trunatula*. We analyzed protein abundance in *M. truncatula* roots after treatment with either 0 or 2.5 mM nitrate in the presence or absence of its symbiont *Sinorhizobium meliloti* after 1, 2 and 5 days following inoculation. Two-dimensional gel electrophoresis combined with mass spectrometry was used to identify 106 differentially accumulated proteins responding to nitrate addition, inoculation or time point. While flavonoid-related proteins were less abundant in the presence of nitrate, addition of Nod gene-inducing flavonoids to the *Sinorhizobium* culture did not rescue nodulation. Accumulation of auxin in response to rhizobia, which is also controlled by flavonoids, still occurred in the presence of nitrate, but did not localize to a nodule initiation site. Several of the changes included defense- and redox-related proteins, and visualization of reactive oxygen species indicated that their induction in root hairs following *Sinorhizobium* inoculation was inhibited by nitrate. In summary, the presence of nitrate appears to inhibit nodulation via multiple pathways, including changes to flavonoid metabolism, defense responses and redox changes.

## 1. Introduction

In response to nitrogen limitation, certain plants, in particular legumes and actinorhizal plants, can form symbioses with nitrogen fixing soil bacteria which convert atmospheric nitrogen into ammonia inside root nodules, which the plant can then convert into amino acids. The legume symbiosis with rhizobia is a well-studied model system for plant symbioses with nitrogen-fixing bacteria. The initially soil-dwelling rhizobia are stimulated by flavonoid exudates from legume roots, which induce the synthesis of Nod factors by rhizobia. Nod factors are necessary for the infection and nodule developmental processes triggered in the root, which lead to the formation of a functional nodule. Rhizobia cause the deformation and curling of root hairs, which then get infected through so-called infection threads. At the same time, initiation of cortical cell divisions lead to the development of a nodule [1]. 

The establishment of the *Rhizobium*-legume symbiosis is regulated by genetic and environmental factors. The numbers of nodules on a legume root system is genetically controlled by an autoregulation mechanism, which limits the numbers of nodules in response to existing nodules on the root system. Mutants defective in autoregulation are super-nodulated. A key gene responsible for autoregulation has been cloned from several legumes and encodes a leucine-rich repeat receptor-like kinase, which acts in the shoot to perceive CLE (CLAVATA3**/**EMBRYO SURROUNDING REGION (ESR)-RELATED) peptides that cause systemic down-regulation of nodulation [2,3]. This gene was named *NARK* (Nodule Autoregulation Receptor Kinase) in soybean, *HAR1* (Hypernodulation Aberrant Root Formation 1) in *Lotus japonicus*, *SUNN* (Super Numeric Nodulation) in *Medicago truncatula* and *SYM29* (Symbiosis 29) in pea [2,3]. 

Nitrogen availability is an important environmental regulator of nodulation [4]. In most legumes, nitrate is a negative regulator of nodulation, although some legumes appear resistant to this negative regulation [5], and low concentrations of nitrogen in form of ammonium can sometime stimulate nodulation [6]. The negative effects of nitrate on nodulation range from effects on the bacterial symbiont to reduced infection and nodule development in the host. For example, nitrate inhibits the synthesis of Nod gene-inducing flavonoids in host roots [7], the expression of the transcription factor NIN (NODULE INCEPTION), which is central to nodulation [8], and can limit the amounts of Nod factors synthesized by rhizobia [9]. Nodule development can be inhibited at different stages during infection, nodule initiation, senescence and nodule functioning, e.g., via inhibition of nitrogenase, the enzyme that converts nitrogen into ammonia [4]. Interestingly, the N source can significantly influence plant responses to nitrogen [10], as well as to Nod factors or rhizobia. For example, while addition of ammonium sources to *L. japonicus* roots inhibited root hair deformation, the addition of nitrate did not reduce this phenotype [8]. Most autoregulation mutants show resistance to nitrate, i.e., they still nodulate, usually above wild type levels, in the presence of otherwise inhibitory levels of nitrate availability, suggesting that nitrate generates a signal that interacts with autoregulation of nodulation [11]. In soybean (*Glycine max*) and *L. japonicus*, nitrate induces a specific CLE peptide that is most likely perceived by the autoregulation receptor-kinase in the root to inhibit nodulation [12,13]. 

The effect of nitrate on nodulation might be mediated through an interaction with plant hormone action. In alfalfa, nitrate availability enhanced the formation of ethylene in the root [14,15]. Ethylene has been shown to restrict nodulation at various stages in *Medicago* sp. [16,17]. Nitrate was also shown to affect levels or signaling of cytokinin [18,19,20], a positive regulator of nodulation [21,22]. Cytokinin is a likely long distance signal relaying information about the nitrogen status of the root to the shoot, and vice versa, which could be important for monitoring and balancing the carbon-nitrogen status of the plant [23]. In *L. japonicus*, nitrate was shown to inhibit the expression of the transcription factor NIN during early nodulation, which acts downstream of cytokinin signaling [8]. Nitrate is also likely to interact with auxin signaling in the root [24] and alters shoot-to-root auxin transport during nodulation in *M. truncatula* [25]. In soybean, rhizobia inoculation led to an increase in auxin concentration in the root system, and this was inhibited in the presence of nitrate [18]. At the stage of nodule growth, sucrose supply from the shoot can be a limiting factor at high nitrate concentrations in soybean [26]. Despite these studies, the cellular and molecular processes targeted by nitrate in the inhibition of nodulation are poorly understood. 

To characterize the global protein changes occurring in response to nitrate during the early stages of nodule formation, we carried out a comparative proteome analysis of *M. truncatula* in the presence and absence of nitrate. While gene expression analyses utilizing microarrays can give information about a larger number of genes than proteome analysis can typically give for proteins, the proteome provides a more accurate picture of the biochemical state of cells and tissues. Protein abundance can be influenced by protein breakdown and modification, in addition to gene expression changes, and therefore transcript and protein abundance do often not correlate well. A root proteome reference map has previously been established [27,28], and nodulation changes were assessed in earlier studies. For example, protein changes were analyzed in soil-grown nodulated roots between two days and six weeks after inoculation [29], in root nodules [30] and in roots of wild type and the ethylene-insensitive *sickle* mutant [31]. In addition, a comparison of proteomes of *M. truncatula* wild type and autoregulation mutant showed extensive overlaps of nodulation and auxin regulated genes [32]. In this report, we are presenting a proteome analysis of *M. truncatula* roots in the presence and absence of nitrate and/or rhizobia over the first five days of nodulation. We followed these changes with a number of metabolite-based assays to verify their involvement in nitrate inhibition of nodulation. 

## 2. Results

### 2.1. Nodulation in the Presence of Nitrate

**Figure 1:**
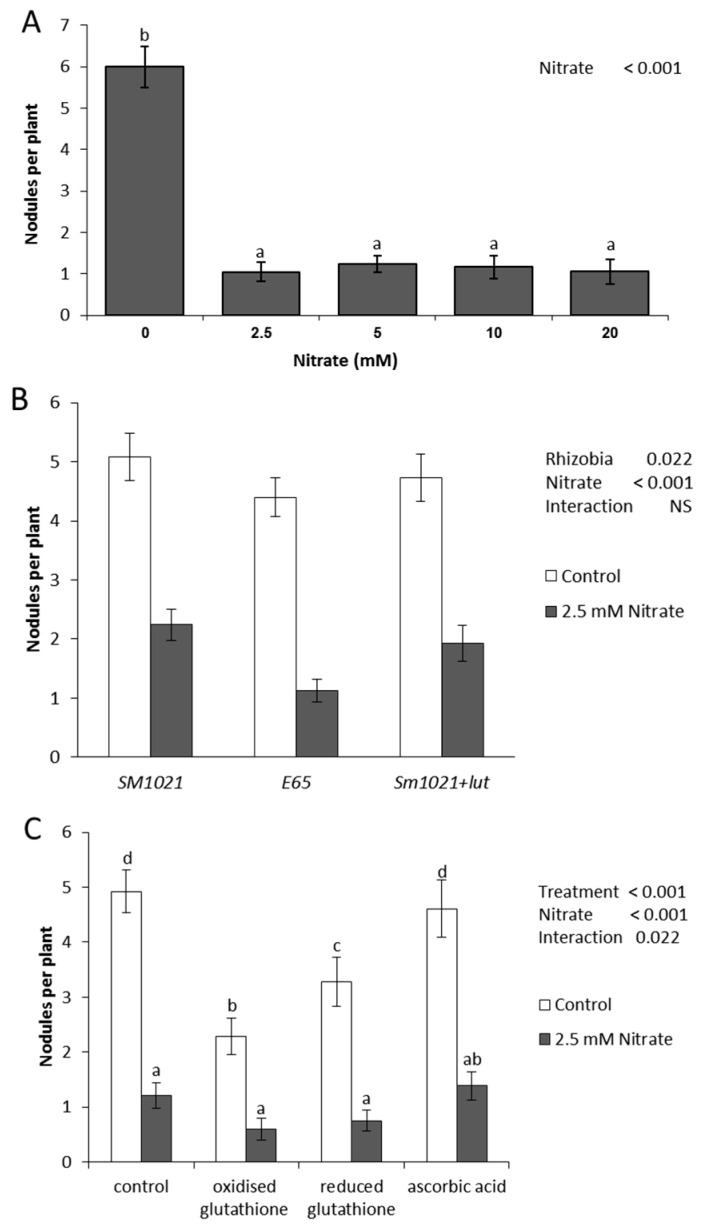
Reduction of nodule numbers in *M. trunatula* in response to nitrate. (**A**) Nodule number responses to varying concentrations of nitrate. The histograms show means of nodule numbers per plant on plates containing the indicated levels of nitrate, three weeks p.i. (post inoculation) with *S. meliloti* (*n* = 21). Error bars represent standard error. Bars marked with different lower case letters show statistically significant differences (*p* < 0.001, one-way ANOVA); (**B**) Nodule number responses to different rhizobia. Plants were nodulated in the absence and presence of 2.5 mM nitrate with either the wild type *S. meliloti* strain (*SM1021*), an *S. meliloti* strain with constitutive nodD2 expression (*E65*) or the *S. meliloti* strain 1021 grown in the presence of the Nod gene inducer luteolin (*Sm1021+lut*). The histograms show means of nodule numbers per plant on plates three weeks p.i. (*n* = 25). Error bars represent standard error. Nitrate (*p* < 0.001) and strains (*p* = 0.022) had a significant effect on nodule numbers (two-way ANOVA); (**C**) Nodule number responses to changes in the redox state. The histograms show means of nodule numbers per plant on plates containing 0 or 2.5 mM of nitrate and 100 µM of either oxidized glutathione, reduced glutathione or ascorbic acid, three weeks p.i. with *S. meliloti* (*n* = 19–26). Error bars represent standard error. Nitrate (*p* < 0.001) and treatments (*p* < 0.001) had a significant effect on nodule numbers, and there was a significant interaction between both at *p* = 0.022 (two-way ANOVA). Bars marked with different lower case letters show statistically significant differences.

To establish the nitrate concentrations that were inhibitory to nodulation in *M. truncatula* under our growth conditions, we grew seedlings in the presence of 0, 2.5, 5, 10 or 20 mM nitrate in the growth medium. A count of nodule numbers after three weeks showed that 2.5 mM nitrate significantly reduced the numbers of nodules per root to under 20% of nodules on roots growing in the absence of nitrate (*p* < 0.05; *n* = 21; one-way ANOVA). Higher concentrations of nitrate did not reduce nodule numbers further (Figure 1A). Therefore, 2.5 mM nitrate was chosen for subsequent proteomics experiments, as we wanted to minimize the nitrate concentration to exclude indirect effects on other growth parameters. At this concentration, nitrate did not significantly affect root length in inoculated or uninoculated roots (*p* > 0.05; *n* = 21; one-way ANOVA, Figure S1). The density of lateral roots was not significantly affected by 2.5 mM nitrate in the absence of rhizobia (*p* > 0.05; *n* = 21; one-way ANOVA, Figure S2), but in the presence of rhizobia nitrate altered lateral root density significantly (*p* < 0.05; *n* = 21; one-way ANOVA, Figure S2). However, the majority of emerged lateral roots were formed in a root zone closer to the root base than the zone of nodule formation that was harvested for proteomics assays.

**Figure 2:**
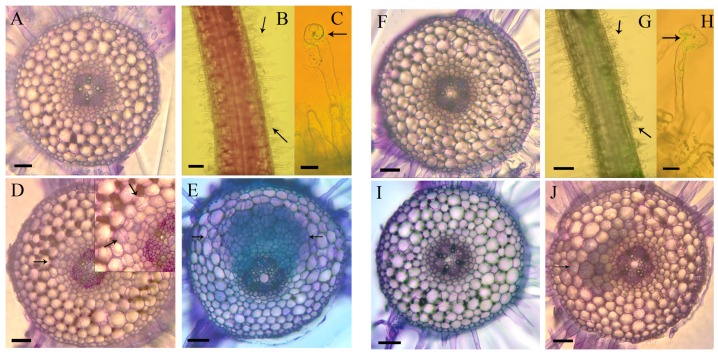
Effect of nitrate on nodulation phenotypes in *M. trunatula*. All pictures show roots or cross sections of roots at the inoculation site stained with Toluidine Blue. Panels **A** to **E** show roots grown in the absence of nitrate, panels **F** to **J** show roots grown on 2.5 mM nitrate-containing medium. (**A**) Uninoculated control root 24 h after mock-inoculation. No root hair curling or cell divisions are visible; (**B**) Root inoculated with *S. meliloti* 24 h p.i. shows a zone of curled and deformed root hairs (between arrows); (**C**) Magnification of a curled root hair, arrow points to the typical Shepard’s crook; (**D**) Root inoculated with *S. meliloti* 48 h p.i. showing the first inner cortical and pericycle cell divisions (arrow). The zone of cell division is enlarged in the insert panel; (**E**) Root inoculated with *S. meliloti* 120 h p.i. showing a developing nodule primordium (between arrows); (**F**) Nitrate treated control root 24 h after mock-inoculation; (**G**) Nitrate treated root 24 h p.i. with *S. meliloti* shows a zone of curled and deformed root hairs (between arrows); (**H**) Magnification of a curled root hair, arrow points to the typical Shepard’s crook; (**I**) Nitrate treated root inoculated with *S. meliloti* 48 h p.i.. No cortical or pericycle cell divisions are visible; (**J**) Nitrate treated root 120 h p.i. with *S. meliloti* showing very few inner cortical and pericycle divisions (arrow). Nine of ten roots at this time point showed no cell divisions. The magnification bars represent 100 µm in **A**, **D**, **E**, **F**, I and J; 400 µm in **B** and **G**; 25 µm in **C** and **H**.

To test whether 2.5 mM nitrate inhibited nodulation at early stages of nodulation, we sectioned ten roots each at 24 h, 48 h and 120 h p.i. (post inoculation). In roots grown in the absence of nitrate, root hair curling was visible in all roots from 24 h p.i. (Figure 2B,C), the first cell divisions in the cortical and pericycle cell layers were seen after 48 h p.i. in eight of ten roots (Figure 2D). Small nodule primordia were visible after five days in nine of ten sectioned roots (Figure 2E). In roots grown in the presence of 2.5 mM nitrate, root hair curling was visible on all roots (Figure 2G,H). No cortical cell divisions were observed within 48 h (Figure 2I). After five days, one of ten roots showed limited cell divisions in the pericycle and inner cortex (Figure 2J), similar to roots of nitrate-deficient roots after 48 h p.i., but no cell divisions were observed in the other nine roots. 

### 2.2. Proteome Analysis

To identify proteins responding to nitrate addition during nodule formation, we carried out a proteome analysis. The experiment involved 12 treatments, including nitrate addition (0 or 2.5 mM), inoculation with *S. meliloti* compared to mock-inoculation, and three time points, 24, 48 and 120 h p.i. Three biological repeats were sampled for each of the 12 treatments, with a pool of 60 root pieces, comprising a 1 cm segment around the inoculation zone, per sample. Silver stained gels showed approximately 2000 proteins per gel (Figure S3), of which 1200 could be matched across all 36 gels. We carried out a three-way analysis of variance (ANOVA) to determine differential accumulation levels of proteins in response to nitrate, inoculation, time point, and interactions between those treatments. Of 1200 displayed proteins, we found 248 proteins that responded significantly (*p* < 0.05) to nitrate addition, inoculation, time point, or interactions between these treatments (Table S1). Most proteins showed a response to nitrate (97), followed by proteins changing across the three time points (96) and proteins being affected by inoculation (38), in addition to protein showing a significant interaction between treatments (Table S1). Many proteins showed responses to more than one treatment.

We identified 106 of the differentially displayed proteins from Coomassie-stained gels of each treatment, after matching each Coomassie gel to the silver-stained gels. Table S2 shows the details of the mass spectrometric identification of these proteins, and Figure S3 depicts a reference map with the location of the identified proteins on a 2DE (2-dimensional gel electrophoresis) reference gel. The 106 proteins matched to 82 different Gene IDs and included 13 occasions in which more than one protein spot matched the same Gene ID. Of the identified proteins, most could be classified as defense or stress responsive (42), followed by primary metabolism (33), protein processing (25), energy metabolism (11), secondary metabolism (11), gene regulation (10), cell structure (7), transport (2) and signaling (1). Another five proteins had unknown functions. Most of the proteins were affected by nitrate treatment (42 proteins) or by time point (51 proteins). Only 11 of the identified proteins responded to inoculation alone, but an additional nine showed an interaction between nitrate and inoculation, and 13 others showed an interaction between inoculation, nitrate and time (Tables S3 and S4). 

There were a large number of proteins responding to nitrate (or nitrate and time point interaction) (Table S3A). These 48 proteins included particularly defense- or stress- related proteins, notably of the disease-resistance response and ABA (abscisic acid)-responsive protein groups, and enzymes involved in primary and energy metabolism. Interestingly, all of the six ABA-responsive proteins and five disease-resistance response protein isoforms were consistently less abundant in the presence of nitrate. Monodehydroascorbate reductase, an enzyme involved in redox control, was strongly induced by nitrate. Two members of the flavonoid pathway, a chalcone reductase and chalcone flavonone isomerase, were less abundant in the presence of nitrate. 

Inoculation of roots with rhizobia induced 38 of the identified proteins, 17 of which showed only an effect of inoculation or an interaction between inoculation and time (Table S3B). These proteins included enzymes of the primary metabolism and the isoflavonoid biosynthesis pathway, a putative transporter, a regulatory protein of the WD40-repeat class, an RNA binding protein, three disease-related proteins, structural proteins (actin and profilin) and several protein processing enzymes. We were particularly interested in the additional 21 proteins that responded to inoculation differently in the presence and absence of nitrate (Table S3C). These proteins included proteins generally down-regulated by rhizobia in the absence of nitrate but induced or unchanged in the presence of nitrate (metallothionein-like protein type 3 (659), an RNA-binding KH domain protein (35), glutathione *S*-transferase (641), pathogenesis-related protein (898), a 20S proteasome subunit (567), tubulin β-1 chain (249), zinc-binding dehydrogenase family oxidoreductase protein (456), ribulose bisphosphate carboxylase/oxygenase activase (401), Kunitz type trypsin inhibitor (840) TGB12K interacting protein (442)), and proteins generally induced by rhizobia in the absence of nitrate but reduced in the presence of nitrate (branched chain amino acid aminotransferase (488), fructose bisphosphate aldolase (355), pyruvate dehydrogenase (414), UTP-glucose-1-phosphate uridylyltransferase (238), triosephosphate isomerase (716), papain family cysteine protease (596), Kunitz type trypsin inhibitor/alpha-fucosidase (874), isoflavone reductase-like protein Bet protein (515), caffeoyl-CoA *O*-methyltransferase (658), chalcone flavonone isomerase (783) and disease-resistance response isoform (922)). In some of these cases, the trend of the nitrate effect additionally depended on the time point. 

Our analysis also revealed 51 identified proteins whose expression did not change in response to nitrate treatment or inoculation, but which showed time dependent expression (Table S3D). These proteins would most likely respond to the different developmental stages of the root from which the samples were collected. Because we harvested a 1 cm root segment surrounding the (mock)-inoculation site, the 24, 48 and 120 h time point samples corresponded to a region just behind the root tip containing parts of the elongation and differentiation zones (24 h p.i.), the differentiation and differentiated root zones (48 h p.i.), and the 120 h samples were harvested from differentiated root regions. Most of the proteins affected by time were enzymes of the primary metabolism and defense- or stress-related proteins, including redox-related proteins. Members of the disease-resistance response protein and ABA-responsive family were generally down-regulated with time, while one polyketide cyclase/dehydrase and lipid transporter (896) was up-regulated and one (978) first up- and then down-regulated. Three proteins involved in glutathione metabolism, glutathione reductase (192), glutathione-*S*-transferase (1129) and hydroxyl-acylglutathione hydrolase (633) showed a general increase in abundance with time.

While the role of many of the identified proteins during nodulation remains unknown, we investigated the involvement of a number of characterized proteins in the nodulation process. These included proteins involved in flavonoid synthesis as well as those involved in defense- and redox-related processes.

### 2.3. Involvement of Flavonoids in Nitrate Inhibition of Nodulation

Nitrate availability was reported to inhibit the synthesis of Nod gene-inducing flavonoids in legumes [7,33]. We partially confirmed this with the reduced abundance of several flavonoid-related enzymes (chalcone reductase, chalcone flavonone isomerase, (iso) flavonoid glucosyltransferase) by nitrate, at least at some of the time points. We hypothesized that *M. truncatula* may fail to nodulate normally due to a lack of Nod gene-induction in its symbiont [7]. To test this, we inoculated plants with (1) the wild type *S. meliloti* strain 1021; (2) the *S. meliloti* strain 1021 grown in the presence of the Nod factor inducer luteolin [34] or (3) an *S. meliloti* strain that constitutively expresses nodD3, i.e., that produces Nod factors in the absence of Nod gene-inducing flavonoids from the legume host [35]. All three strains nodulated *M. truncatula* equally well in the absence of nitrate (Figure 1B). In the presence of 2.5 mM nitrate *M. truncatula*, neither addition of luteolin nor inoculation with a flavonoid-independent *S. meliloti* strain increased nodule numbers beyond the control, rejecting the hypothesis that *M. truncatula* is unable to be successfully nodulated in the presence of nitrate solely due to a lack of Nod gene-induction in its symbiont. 

**Figure 3:**
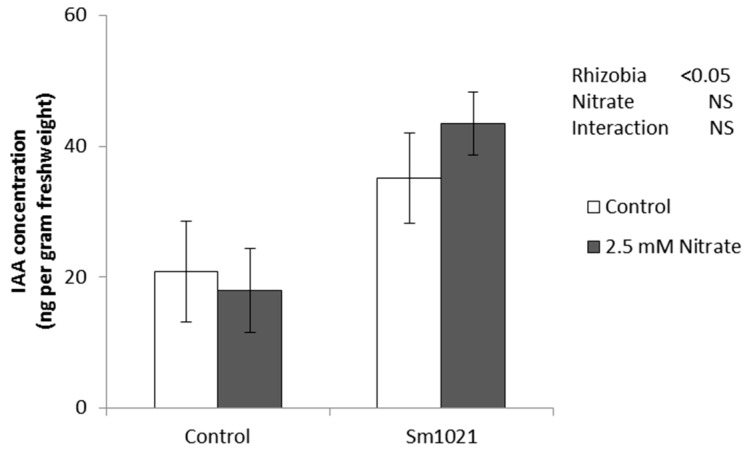
Quantification of auxin in roots in response to nitrate and *S. meliloti* treatment. Indoleacetic acid (IAA) quantification in 1 cm root segments surrounding the inoculation site at 24 h p.i. The histograms show the amount of auxin in mock-inoculated (Control) and *S. meliloti*-inoculated roots in the absence and presence of 2.5 mM nitrate. Two-way ANOVA showed a significant effect of inoculation (*p* < 0.05), but not of nitrate (not significant, NS). Error bars represent standard error. Each measurement is comprised of five biological repeats with at least 30 root segments each.

Flavonoids are also essential for the auxin accumulation at the site of nodule formation early during the nodulation process [36]. In wild type roots, auxin concentrations increase at the inoculation site and auxin maxima are formed at the site of nodule initiation within 48 h p.i., and this requires flavonoids [36]. Quantification of the indoleacetic acid (IAA) contents in *M. truncatula* roots 24 h after inoculation with *S. meliloti* strain 1021 in the presence or absence 2.5 mM nitrate showed that the concentration of auxin increased significantly (*p* < 0.05) in response to inoculation with rhizobia in both treatments compared to the mock-inoculated roots (Figure 3). This suggested that nitrate did not prevent the increase in IAA content at the inoculation site, at least at this time point.

**Figure 4:**
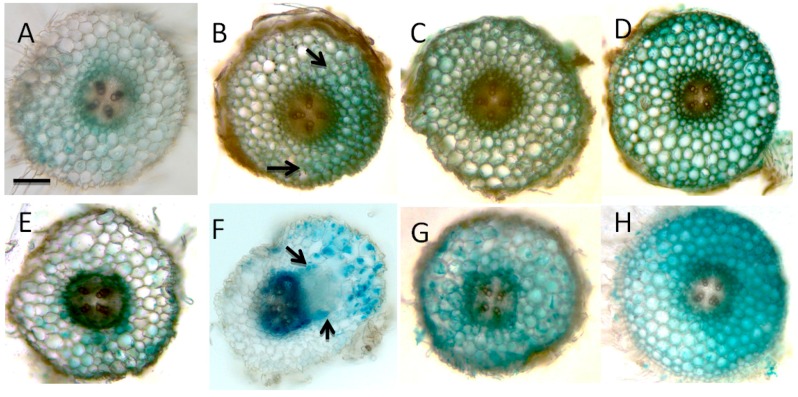
*GH3:GUS* expression indicating auxin responses to nitrate and *S. meliloti* treatment. Sections in **A**–**D** show cross sections through the inoculation site at 24 h p.i., sections **E**–**H** at 48 h p.i. (**A**,**E**) Mock-inoculated roots in the absence of nitrate; (**B**,**F**) *S. meliloti* (1021)-inoculated roots in the absence of nitrate; (**C**,**G**) Mock-inoculated roots in the presence of 2.5 mM nitrate; (**D**,**H**) *S. meliloti* (1021)-inoculated roots in the presence of 2.5 mM nitrate. Pictures are representative of at least ten analyzed roots for each treatment. Arrows in **B** indicate the localized increase in auxin response to *S. meliloti*, arrows in **F** indicate a nodule primordium with localized *GH3:GUS* expression at the base of the primordium and in cortical cells overlying the primordium, whereas *GH3:GUS* expression in the presence of nitrate was more diffuse in the cortex. Magnification bar represents 200 µm.

Localization of auxin responses using the *GH3* promoter showed that nitrate-treated roots generally showed a stronger auxin response in the cortex than no-nitrate treated roots. In response to rhizobia, auxin responses increased in nitrate-treated roots in response to rhizobia at 24 and 48 h p.i. diffusely in the cortex, however, the nitrate-treated roots failed to form a defined auxin maximum or cortical cell divisions (Figure 4). Additional photos of whole-mounted roots are shown in Figure S4 to indicate the range of responses seen in individual roots.

### 2.4. Involvement of Reactive Oxygen Species in Nitrate Inhibition of Nodulation

**Figure 5:**
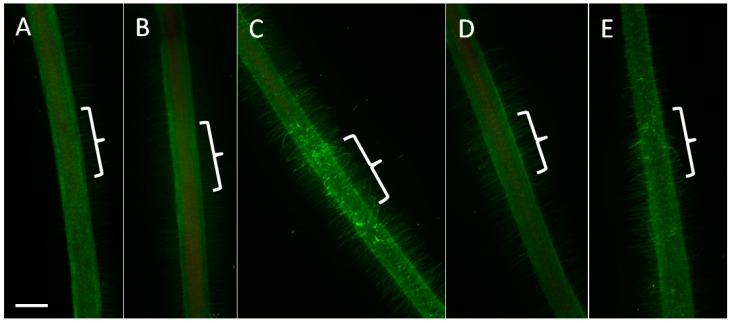
Induction of reactive oxygen species in response to nitrate and *S. meliloti*. Reactive oxygen species (ROS) were visualized at 24 h p.i. following spot inoculation of the root with *S. meliloti* (1021) (marked with a bracket). (**A**) Mock-inoculated root in the absence of nitrate; (**B**) Mock-inoculated root in the presence of 2.5 mM nitrate; (**C**) *S. meliloti* (1021)-inoculated root in the absence of nitrate. Note the strong induction of green fluorescence in root hairs indicating the presence of (ROS) (18/22 roots); (**D**,**E**) *S. meliloti* (1021)-inoculated root in the presence of 2.5 mM nitrate. The lack of response in **D** was found in 22/28 roots, the weak response in **E** was found in 6/28 roots. Magnification bar represents 1 mm.

We observed a large number of changes in protein abundance indicating that nitrate interfered with the disease-response proteins and proteins involved in formation of reactive oxygen species (ROS), e.g., several ABA-responsive and disease-resistance response proteins and monodehydroascorbate reductase (Table S3A). Reactive oxygen species could play an important part in nodule infection and the control of early defense responses to rhizobia [37]. We visualized ROS formation during the early infection process by staining with the ROS-sensitive dye 5- (and 6-) chloromethyl-2’-7’-dichlorodihydrofluorescein diacetate [38] (Figure 5). Roots spot-inoculated with a small, localized drop of rhizobia showed induction of ROS in root hairs in the absence of nitrate within 24 h p.i. (18/21 roots) (Figure 5C). However, in the presence of 2.5 mM nitrate, most roots (22/28) showed no induction of ROS at the inoculation site (Figure 5D). Only 6/28 analyzed roots showed a ROS response, however, this was weaker than in no-nitrate treated roots (Figure 5E). To test whether an imbalance of ROS could be a reason for the failure to nodulate in nitrate-treated roots, we supplemented the roots with oxidized glutathione, reduced glutathione or ascorbic acid. Oxidized glutathione was predicted to lead to an increase in ROS, while reduced glutathione and ascorbic acid were predicted to reduce the availability of ROS [39]. Our results showed that while both oxidized and reduced glutathione reduced nodule numbers in control plants, none of the treatments showed significant rescue of nodulation in the presence of 2.5 mM nitrate (Figure 1C).

## 3. Discussion

Our study aimed at identifying proteins and their associated functions during nodulation that are changed during the early stages of symbiosis, but are altered in expression in the presence of nitrate. The application of 2.5 mM or higher concentrations of nitrate to *M. truncatula* plants was sufficient to reduce nodule numbers to less than 20% of those in control plants. This compares with levels of between 2 and >10 mM nitrate inhibiting nodulation in other legumes [14,40,41]. The effects of nitrate on nodulation were reported to be varied, ranging from loss of infection to the inhibition of nodule initiation and nitrogen fixation, and depend on the level of nitrate applied, time and place of application, growth conditions and plant species [4]. In our study, sectioning of the inoculation site showed that nitrate addition inhibited the initiation of cortical cell divisions, while root hair curling could still be observed, similar to results found in *L. japonicus* [8]. There were no observable cytological differences between nitrate-treated and untreated roots within 24 h of inoculation, whereas at two and five days after inoculation, cell divisions had occurred predominantly in plants growing in the absence of nitrate.

A comparison of protein changes in response to rhizobia with those of other studies showed that some, but not all, proteins responding to *S. meliloti* infection also showed similar changes in transcription. For example, a study by Lohar and colleagues [42] showed similar responses to rhizobia for chalcone flavonone isomerase (783, induced at 24 h p.i.), Transducin/WD40 repeat protein (491, induced at 24 h p.i.), isoflavone reductase (541, reduced at 48 h p.i., although different pattern at 24 h p.i.), Profilin-1 (1040, reduced at 48 h p.i.), fructose bisphosphate aldolase (355, induced at 24 h p.i.), pyruvate dehydrogenase (414, induced at 24 h p.i.), pathogenesis-related protein (875, reduced at 24 and 48 h p.i.), caffeoyl-CoA-*O*-methyltransferase (658, induced at 24 h p.i.), chalcone flavanone isomerase (783, induced at 24 h p.i.), and a methalloprotein-like protein (408, reduced at 24 h p.i.). Many other genes were regulated by rhizobia in that study but these were not identified in our proteomics study. 

The proteomics results showed the most extensive responses of the root to the addition to nitrate (179 of 1200 analysed proteins), while most of them (124) did not vary with inoculation and are likely to be general responses to nitrate. Nitrate can act as a signal as well as a nutrient in roots [43], and nitrate addition to plants can lead to changes in plant growth and architecture, in particular by affecting lateral root initiation and elongation [23,25,44]. In our study, no significant changes in root length were observed in the presence of 2.5 mM nitrate, and lateral root density was not affected by this concentration of nitrate in the absence of rhizobia, although a nitrate concentration-dependent change was found in inoculated roots. Transcriptomics experiments in *L. japonicus* [45] and *M. truncatula* [46] found large numbers of genes affected by the addition of nitrate to plants, including changes to transporters responsible for nitrate uptake and transport, energy metabolism, starch mobilization and organic acid synthesis, for later conversion into amino acids. We found the expression of two amino acid metabolism enzymes (glutamate dehydrogenase (334) and thiosulfate sulfurtransferase (571)) to be induced by nitrate, indicating that nitrate addition could lead to increased synthesis of amino acids. Glutamate dehydrogenase is one of the major enzymes involved in the primary assimilation of nitrate into amino acids [47]. Other changes in the primary metabolism in response to nitrate were less consistent, but in general, enzymes of the glycolysis pathway and starch synthesis were more abundant in nitrate treated roots (Table S3). Glycolysis end products are necessary for organic acid and subsequent amino acid synthesis; therefore, enhanced glycolysis might feed into enhanced amino acid synthesis in nitrate treated roots. Up-regulation of enzymes involved in energy metabolism, including three F1-ATPases, could reflect increased metabolic activity due to increased nitrogen assimilation in nitrate treated roots.

A very consistent response to nitrate was the repression of 11 protein isoforms encoding disease-resistance response proteins and ABA-responsive proteins, two related classes of proteins previously found to be involved in plant defense against pathogens that are activated in response to a range of abiotic stresses [48,49]. Subsequent studies have shown that disease-resistance response proteins and ABA-responsive proteins are also altered in response to symbionts [31,32] and during plant development and protoplast differentiation [50,51,52]. Possible developmental roles of these proteins could be due to the ability of disease-resistance response (PR10) proteins to bind cytokinin (zeatin), steroids (including brassinosteroids), and flavonoids [53]. PR10 promoter activity has also been linked to oxidative stress in plants [54], and it is possible that various biotic and abiotic stimuli that alter PR10 expression act partially by altering the cellular oxidation or redox state. Our study showed evidence of changes in the redox state of the root in response to nitrate, as the induction of ROS by rhizobia was only observed in the absence of nitrate (Figure 5). While the exact role of ROS during nodulation is not clear, it could indicate an initial defense response to rhizobia and/or participate in cell wall crosslinking during root hair curling or infection thread formation [37,55,56]. If formation of ROS is part of a defense response to rhizobia, the reduced induction of ROS in the presence of nitrate is consistent with the reduced accumulation of several disease-resistance proteins in the presence of nitrate. It remains to be tested why such a reduction of defense responses would prevent nodule development.

Previous studies in *M. sativa* suggested that nitrate has an effect on the induction of ethylene by rhizobia. Ethylene, like high nitrate availability, is a negative regulator of nodulation and might mediate defense responses [17]. Prayitno and colleagues [31] identified several ethylene-induced proteins in *M. truncatula* roots that are altered during nodulation. Proteins inducible by ethylene and/or requiring ethylene signaling included an ABA-responsive protein, heat shock proteins, Kunitz proteinase inhibitor, ascorbate peroxidase and ACC (aminocyclopropane-1-carboxylic acid) oxidase. Our study found several isoforms of Kunitz proteinase inhibitor (KPI) and ABA-responsive proteins to be altered by nitrate after inoculation. KPI was identified as a major apoplastic protein in soybean [57] and in *M. truncatula* suspension cultures [28], and might be involved in preconditioning cells during defense responses [58]. These protein changes could thus indicate altered ethylene-dependent defense or stress responses as a result of nitrate treatment. Future experiment could test whether nitrate altered ethylene signaling in *M. truncatula* during early nodule development. 

Nitrate availability was also reported to have a negative influence on the synthesis of Nod gene-inducing flavonoids in legumes [7]. In this study, we found that chalcone reductase (532), acting at the base of the isoflavonoid and aurone synthesis pathways, was initially reduced by rhizobia in the absence, but induced in the presence of nitrate. At later time points this trend was reversed. A chalcone flavonone isomerase (783) was induced by rhizobia in the absence of nitrate but repressed in the presence of nitrate. Chalcone flavonone isomerase leads to the synthesis of naringenin and liquiritigenin, which form the precursors for flavones, dihydroflavonols, flavonols, anthocyanins and isoflavonoids [59]. Isoflavone reductase (515), which leads to the synthesis of isoflavonoid end products in *M. truncatula*, was similarly repressed by rhizobia in the presence and absence of nitrate. In addition, strong induction of a WD40 repeat protein could be linked to the regulation of flavonoid synthesis genes, as a WD40 protein interacts with other transcription factors in the control of the flavonoid pathway [60,61]. Since one role of flavonoid metabolites during nodulation is the induction of Nod genes in rhizobia [34], we tested whether supplementation of rhizobia with the Nod gene-inducing flavonoid luteolin, or inoculation of plants with rhizobia that are independent of flavonoids for Nod gene activation, could alleviate the inhibition of nodulation by nitrate. However, we found that this was not the case (Figure 1B), indicating that lack of flavonoid Nod gene-inducers by itself may not a sufficient reason for nodule inhibition by nitrate.

Flavonoids also control auxin transport and accumulation during nodulation in *M. truncatula* [36,62], and nitrate was found to interfere with auxin accumulation in soybean plants following inoculation with rhizobia [18]. Auxin was also shown to alleviate some of the negative effects of nitrate on nodulation [63] and in Arabidopsis, auxin signaling is required for mediating the effect of nitrate on root branching [24]. Therefore, we tested if nitrate-treated plants were deficient in auxin accumulation or response during the early stages of nodulation. This was determined through direct measurements of the auxin IAA, auxin responses determined through proteome analysis and localization of auxin responses using *GH3:GUS* reporter plants. Direct measurement of IAA content (Figure 3) showed no significant changes in response to nitrate within 24 h at the inoculation site, where an increase in auxin concentration following rhizobia inoculation had previously been established [36]. This is in contrast to the inhibition of auxin increases in inoculated soybean roots by nitrate [18], however, in that study hormone measurements were made at different time points and different locations in the root system.

Some of the proteins altered by nitrate during nodulation were previously shown to be auxin-inducible in *M. truncatula*. Van Noorden et al. [32] identified proteins in *M. truncatula* roots responding to auxin treatment and to inoculation with rhizobia. Of 21 identified proteins with altered expression patterns in response to nitrate and inoculation (Table S3), seven (monodehydroascorbate reductase, fructose bisphosphate aldolase, a disease-resistance response protein isoform, quinone oxidoreductase, triosephosphate isomerase, tubulin-b-2 chain and UDP-glucose pyrophosphorylase) were auxin-inducible and one (glyceraldehyde-3-phosphate dehydrogenase) auxin-repressed [32]. This may reflect some subtle changes to auxin perception in the presence of nitrate. Localization of auxin responses in *GH3:GUS* transgenic plants indicated that auxin responses were increased in nitrate-treated plants, but that no auxin maxima were formed, which are likely a prerequisite for nodule initiation [36]. While it is currently unknown which exact flavonoid metabolite orchestrates auxin transport control and local auxin accumulation during nodule initiation, once this compound is identified it would be interesting to test whether its abundance is reduced by nitrate.

## 4. Materials and Methods 

### 4.1. Plant Growth

Seeds of *Medicago truncatula* cv. Jemalong A17 were surface sterilized with 6% (*w*/*v*) sodium hypochlorite, washed eight times in sterile distilled water and transferred to water agar plates. After a two day period in the dark at 4 °C, plates were transferred to 28 °C over night to germinate. Seedlings of approximately 1 cm length were transferred to 15 cm diameter Petri dishes containing Fåhraeus medium [64] without the inclusion of the ethylene inhibitor AVG (aminoethoxyvinylglycine), and incubated in a controlled growth chamber at 21 °C, with a light intensity of approximately 100 µmol·m^−2^·s^−1^, and 16 h of light per day. The Fåhraeus plates contained either no source of nitrogen or KNO_3_ at 2.5 mM, 5 mM, 10 mM or 20 mM, where appropriate, with controls receiving KCl at the same concentration. The effect of the potassium in the nitrate source as opposed to other nitrate sources, e.g., calcium nitrate, was previously shown not to affect nodulation results [40]. For experiments testing the effect of redox compounds on nodulation, 100 µM of oxidized glutathione, reduced glutathione or ascorbic acid (all from Sigma-Aldrich, Sydney, Australia) were incorporated in the agar from the first day after seedling germination. Plants were grown under sterile conditions vertically on the surface of the agar plates and the bottom half of the plates was covered in black paper to minimize light exposure to the roots. After three days, the roots had reached a length of approximately 4–5 cm and were inoculated with *S. meliloti*. For experiments shown in Figure 1, where total nodule numbers were counted on the root system, the roots were flood-inoculated with 10 µL of bacterial culture spread along the root. For microscopy (Figure 2) and proteomics experiments, where a localized infection was desired, a 5 µL drop of *S. meliloti* at the zone of emerging root hairs. *S. meliloti* strain 1021 was grown as an over night culture in liquid Bergensen’s Modified Medium (BMM) at 28 °C overnight, and diluted with sterile water to an OD_600_ of 0.1. As a control, the roots were inoculated with an equivalent amount of diluted BMM. The inoculation site was marked on the plate. For nodulation assays, seedlings were grown for three weeks and nodules counted on each root under a stereomicroscope. Where indicated, *S. meliloti* cultures were supplemented with 2 µM luteolin (Sigma-Aldrich) over night to induce Nod gene expression.

### 4.2. Microscopy

To follow the extent of nodule development in roots in the presence and absence of nitrate, the 10 mm region spanning the inoculation site was embedded in 3% DNA grade agarose (Progen Biosciences, Sydney, Australia) and sectioned on a Vibratome (1000 Plus, Vibratome Company, Saint Louis, MO, USA) at 100 µm thickness. Sections were mounted on glass slides in water and examined with a Leica DMBL microscope (Leica Microsystems GmbH, North Ryde, Australia) and photographed with a mounted SPOT RT slider CCD camera (Diagnostic Instruments, Sterling Heights, MI, USA). Sections were stained with Toluidine Blue by immersing sections for 30 s in 0.5% Toluidine Blue (pH 4.4) and rinsing in distilled water.

### 4.3. Proteome Analysis

After 24, 48 and 120 h p.i., root segments of 10 mm length spanning the inoculation site were excised with a sterile scalpel blade and frozen in liquid nitrogen within 30 s. The experiment included 12 treatments (roots inoculated or mock-inoculated at 24, 48 and 120 h p.i. in both presence and absence of 2.5 mM nitrate). The treatments were repeated three times in total, resulting in 36 biological samples. Each of the 36 samples consisted of 60 root segments, which were pooled before protein extraction. Each sample was used to produce one silver stained analytical 2D gel. In addition, for each of the 12 treatments, 120 root segments were combined and extracted for a preparative Coomassie gel. For silver stained gels a total of 200 µg of protein was loaded, for a Coomassie stained gel 1000 µg were loaded. If gels showed any separation or staining artifacts they were discarded and repeated with a new sample.

Protein extraction was done subsequent to a trichloroacetic acid/acetone precipitation as reported previously [27]. Proteins were separated in the first dimension on precast immobilized pH gradient (IPG) gel strips of 24 cm length with a linear pH gradient of 4 to 7 (GE Healthcare, Uppsala, Sweden). Second dimension separation took place on precast Excel Gels with a 12%–14% acrylamide gradient (GE Healthcare). The proteins on analytical gels were visualized by silver staining or by Colloidal Coomassie staining [27]. 

Gels were scanned at 600 dots per inch resolution using a UMAX Powerlook III scanner (UMAX Techologies, Freemont, CA, USA). All spot detection and spot comparisons were made using Image Master 2D Platinum version 5.0 (GE Healthcare, Paramatta, Australia). This included matching between silver-stained and Coomassie-stained gels. The relative molecular mass of proteins was determined by the co-migration of protein standards (Low Molecular Weight Markers, GE Healthcare) using Image Master 2D Platinum version 5.0 (GE Healthcare).

For the quantification of spot volumes across the different treatments, silver-stained gels were used. To reduce gel-to-gel variation, the spot volumes were normalized by calculating their relative spot volumes (% spot volume/total spot volumes of all proteins on the gel) using Image Master 5.0 software (GE Healthcare). Statistical analysis was carried out for each of approximately 1200 protein spots matched across all gels. The treatments tested in this study were: (1) nitrate addition (0 or 2.5 mM); (2) inoculation with rhizobia (control or *Sinorhizobium* inoculation); and (3) the time point of inoculation (24, 48 or 120 h p.i.). Each factor was analyzed separately, and subsequently the interaction of nitrate x inoculation, nitrate x time, inoculation x time and nitrate x inoculation x time was analyzed. Data were tested for normality by Shapiro-Wilk test, and when the data were not normally distributed, a non-parametric test (Kruskal-Wallis test) was used to test for significant differences of the treatment. For normally distributed data with homogenous variance, three-way analysis of variance (ANOVA) using restricted maximum likelihood method (REML) was calculated to test significant difference within and between treatments at *p* < 0.05. The least significant difference (LSD) was used as a post hoc test when the three-way ANOVA showed significance. All statistical analyses were carried out using GenStat for Windows (Edition 5.0).

Protein spots showing differential expression were manually excised from Coomassie-stained gels using sterile scalpel blades. The gel pieces were destained in 50% 25 mM ammonium bicarbonate, pH 7.8, 50% acetonitrile, dehydrated with 100% acetonitrile, and digested with 8 µL of 15 ng/µL sequencing grade modified trypsin (Promega, Madison, WI, USA) in 25 mM NH_4_HCO_3_, pH 7.8 for 16 h at 37 °C. The resulting peptides were acidified with 8 µL of 1% trifluoroacetic acid (TFA) and extracted by sonication. Peptides that were faint on the gels or did not initially produce strong spectra were purified using C_18_ reversed-phase ZipTips (Millipore Corp., Bedford, MA, USA) following the manufacturer’s instruction. Peptides were eluted with 70% acetonitrile in 0.1% TFA for MALDI-TOF(-TOF) analysis.

For MALDI-TOF(-TOF) analysis, a 1 µL sample aliquot was spotted onto a sample plate, which was pre-spotted with 1 µL of matrix (8 mg/mL α-cyano-4-hydroxycinnamic acid in 70% *v*/*v* acetonitrile and 1% TFA) and allowed to air dry. MALDI TOF analysis was performed with an Applied Biosystems 4700 Proteomics Analyser (at the Australian Proteome Analysis Facility, Sydney, Australia). MALDI-TOF-TOF analysis was done with an Applied Biosystems 4800 Proteomics Analyser (Applied Biosystems, Foster City, CA, USA) with TOF/TOF optics in MS mode. The spectra were acquired in reflectron mode over the *m*/*z* range of 800–3500 Da. The instrument was then switched to MS/MS mode where the 25 strongest peptides from the MS scan were isolated and fragmented and their mass and intensities were measured. A near point external calibration was applied and gave a typical mass accuracy of ~50 ppm or less. 

Mass spectra and ion data generated by MALDI TOF and MALDI TOF MS/MS were used to search for protein identification against the *M. truncatula* EST (Expressed Sequence Tag) database [65] using Mascot Daemon version 2.1.0 software program (Matrix Science, Boston, MA, USA). We then used the *Medicago truncatula* Gene Expression Atlas [66] to match the TCs against the *M. truncatula* gene identifiers. For peptide matching, a maximum of one mis-cleavage per peptide, and peptide modifications by oxidation of methionine and carbamidomethylation of cysteine were allowed. The peptide mass tolerance and ion mass (MS/MS) accuracy used for peptide matching were set at 100 ppm and 0.4 Da, respectively. The confidence of peptide matches was based on the significant value of the MOWSE score, the mass accuracy, number of peptide matches and the percentage of sequence coverage. For the functional classification of proteins, we followed [67], but classified proteins involved in glucose metabolism as “Primary Metabolism”, and combined “Protein Synthesis” and “Protein Destination and Storage” into “Protein Processing”. Putative protein function was assessed after interrogation of the *M. truncatula* EST annotations, the KEGG and Swiss Prot databases [68,69], as well as reports from the literature. 

### 4.4. Histochemical GUS Staining

Histochemical GUS (β-glucuronidase) staining using stably transformed *M. truncatula* plants expressing the *GH3:GUS* gene was performed as described in [32].

### 4.5. Quantification of Auxin

*Medicago truncatula* seedlings were grown as described above. *M. truncatula* seedlings were subjected to four treatments: (a) grown on media with/without 2.5 mM KNO_3_ (2.5 mM KCl as control treatment); (b) with/without spot inoculation with *S. meliloti* strain 1021. Four-day-old seedlings were spot-inoculated with either BMM (control treatment) or *S. meliloti* at the nodulation susceptible zone (~5 mm behind the root tip). At 24 h p.i., root segments of 5 mm length surrounding the inoculation spot were excised, placed in a pre-weighed 2 mL Safe-Lock Eppendorf tube and immediately snap frozen in liquid nitrogen. Root tissues from five biological replicates were harvested, with each replicate consisting of at least 30 root segments. Tubes containing the root samples are weighed and stored at −80 °C until they were ready to be processed for liquid chromatography coupled to a quadrupole time-of-flight tandem mass spectrometer, as described in [36]*.*

### 4.6. Staining of Reactive Oxygen Species

Roots were excised 24 h after spot-inoculation with *S. meliloti* strain 1021 or mock-inoculation with BMM, and submerged in a solution containing 10 µM 5- (and 6-) chloromethyl-2’-7’-dichlorodihydrofluorescein diacetate, acetyl ester (CM-H_2_DCFDA, Molecular Probes) for 1 h. CM-H_2_DCFDA was dissolved in dimethyl sulfoxide (DMSO) at 100 mM and then diluted into distilled water before staining. Following the staining, roots were immersed in distilled water for 30 min to rinse off excess stain, then mounted on glass microscope slides in distilled water. Green fluorescence indicating the formation of ROS was visualized under a Leica M205 FA stereomicroscope ET Blue LP filter system (max. excitation at 470 nm with a 515 nm long pass filter, (Leica Microsystems GmbH, North Ryde, Australia)). Images were taken with a Leica DFC550 high-speed digital camera (Leica Microsystems) using identical exposure settings for each root to allow qualitative comparisons. At least 20 roots were examined for each treatment.

## 5. Conclusions

We found that the presence of nitrate inhibited nodule initiation in *M. truncatula*, and that this was accompanied by changes in multiple metabolic and signaling pathways. While flavonoid enzymes and enzymes involved in defense and redox control were strongly altered in the presence of nitrate, neither provision of flavonoids to rhizobia nor changes to the internal ROS balance alleviated the reduction of nodule numbers by nitrate. Thus, nitrate is likely to interfere with multiple processes at the same time to inhibit nodulation.

## Supplementary Materials

Supplementary materials can be found at http://www.mdpi.com/1422-0067/17/7/1060/s1.
