# Supplementary material for: Molecular Signals Controlling the Inhibition of Nodulation by Nitrate in Medicago truncatula
Source: Int J Mol Sci. 2016 Jul 2;17(7):1060. doi: 10.3390/ijms17071060 (PMC4964436; doi:10.3390/ijms17071060)
Supplement: Supplementary file 1 [file ijms-17-01060-s001.zip › ijms-134001-Supplementary Materials/ijms-134001-supp-final.pdf]

Supplementary Materials: Molecular Signals Controlling the Inhibition of Nodulation by Nitrate in *Medicago truncatula*

Giel E. van Noorden, Rob Verbeek, Quy Dung Dinh, Jian Jin, Alexandra Green, Jason Liang Pin Ng and Ulrike Mathesius

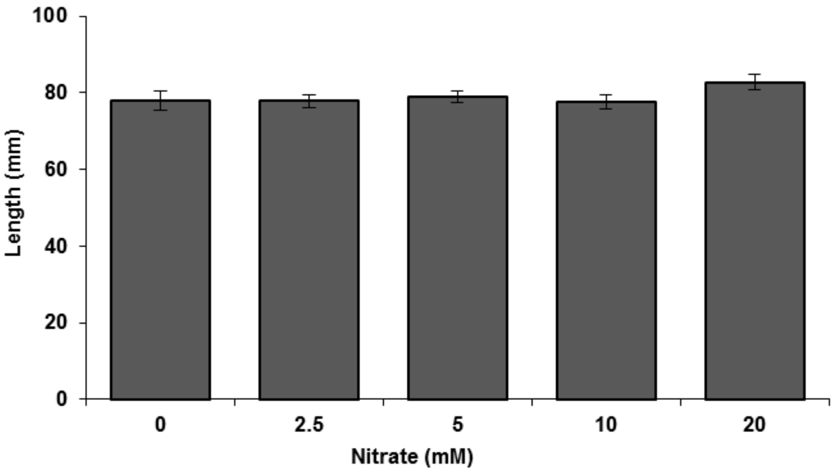

(**A**)

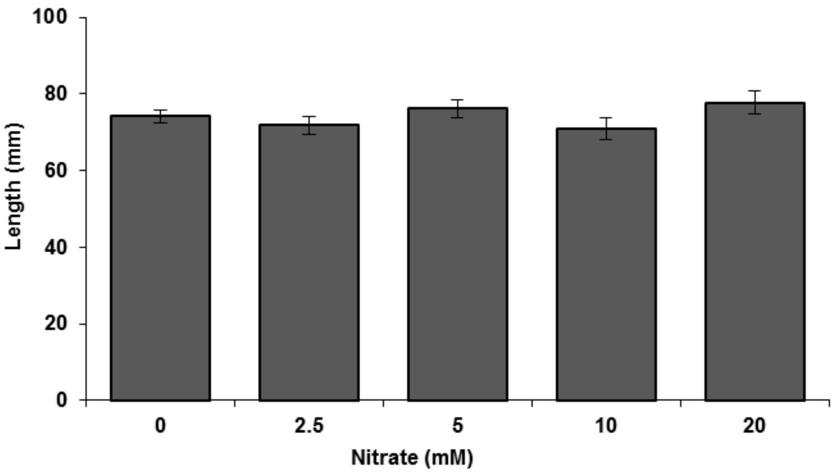

(**B**)

Figure S1. Root length of *Medicago truncatula* seedlings gown in the presence nitrate in the growth medium. (**A**) Uninoculated roots; (**B**) Roots inoculated with *S. meliloti* strain 1021. There were no significant differences in root length in response to nitrate ($p > 0.05$; one-way ANOVA).

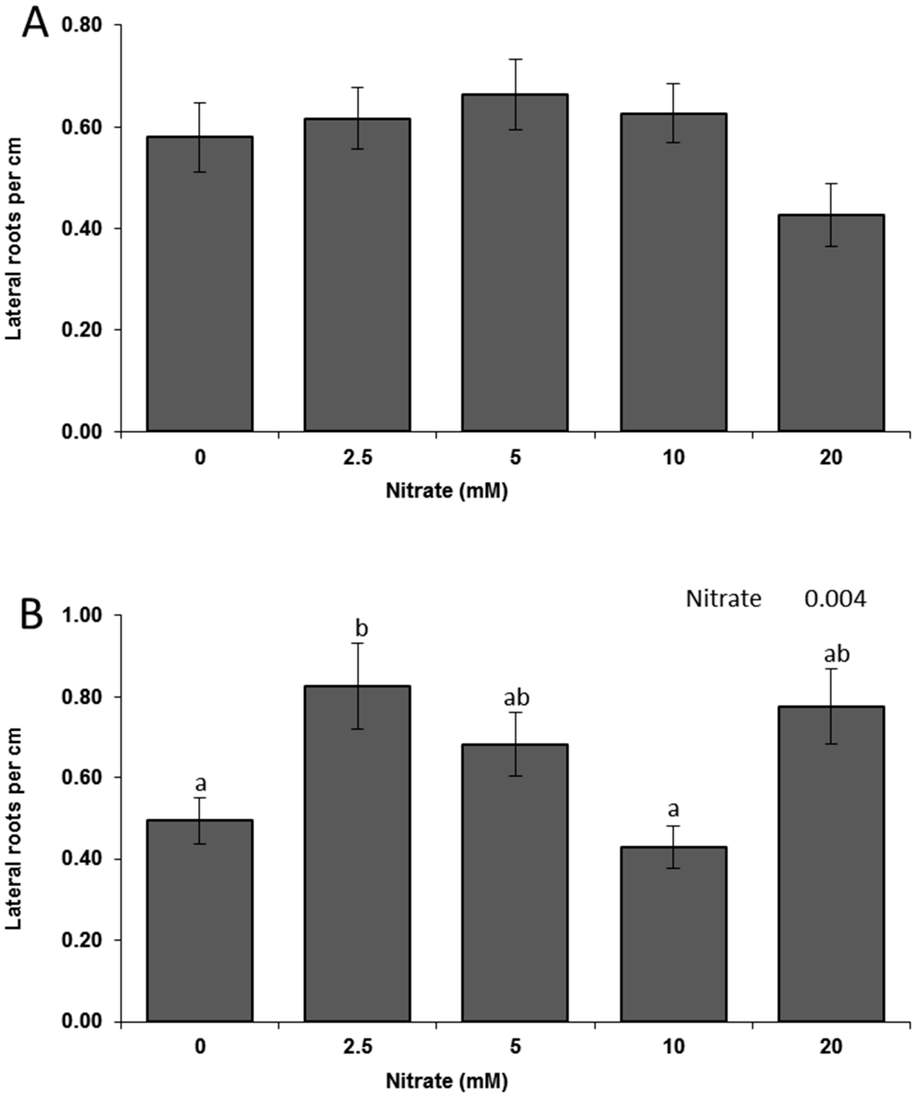

Figure S2. Lateral root density of *Medicago truncatula* seedlings gown in the presence nitrate in the growth medium. (**A**) Uninoculated roots; (**B**) Roots inoculated with *S. meliloti* strain 1021. There were no significant differences in root length in response to nitrate in the uninoculated roots, but a significant effect ($p < 0.005$) in the inoculated roots (one-way ANOVA). Treatments labelled with different lower case letters differ significantly at $p < 0.05$.

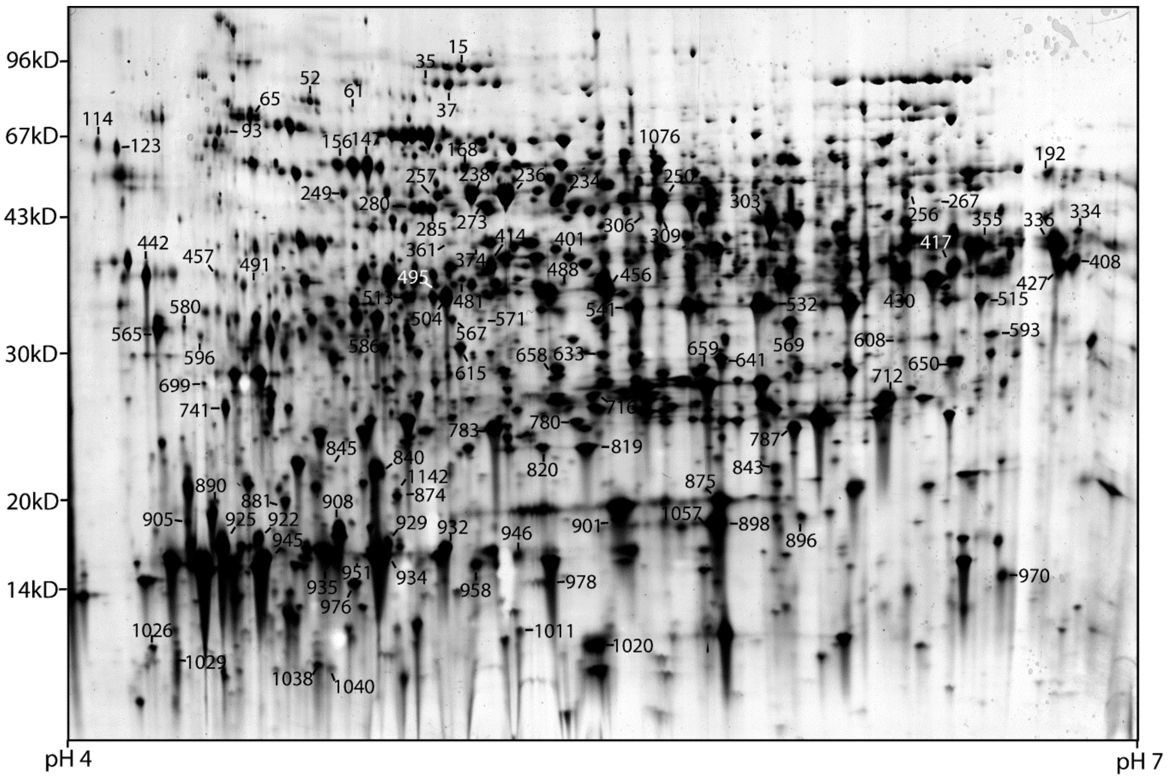

Figure S3. Proteome reference map. The gel shows the silver-stained reference gel used for matching of gel images. Proteins were resolved between approximately 10 and 100 kDa between p*I*s of 4 and 7. Differentially displayed proteins identified in this study are numbered. The numbers refer to proteins listed in Tables S2–S4.

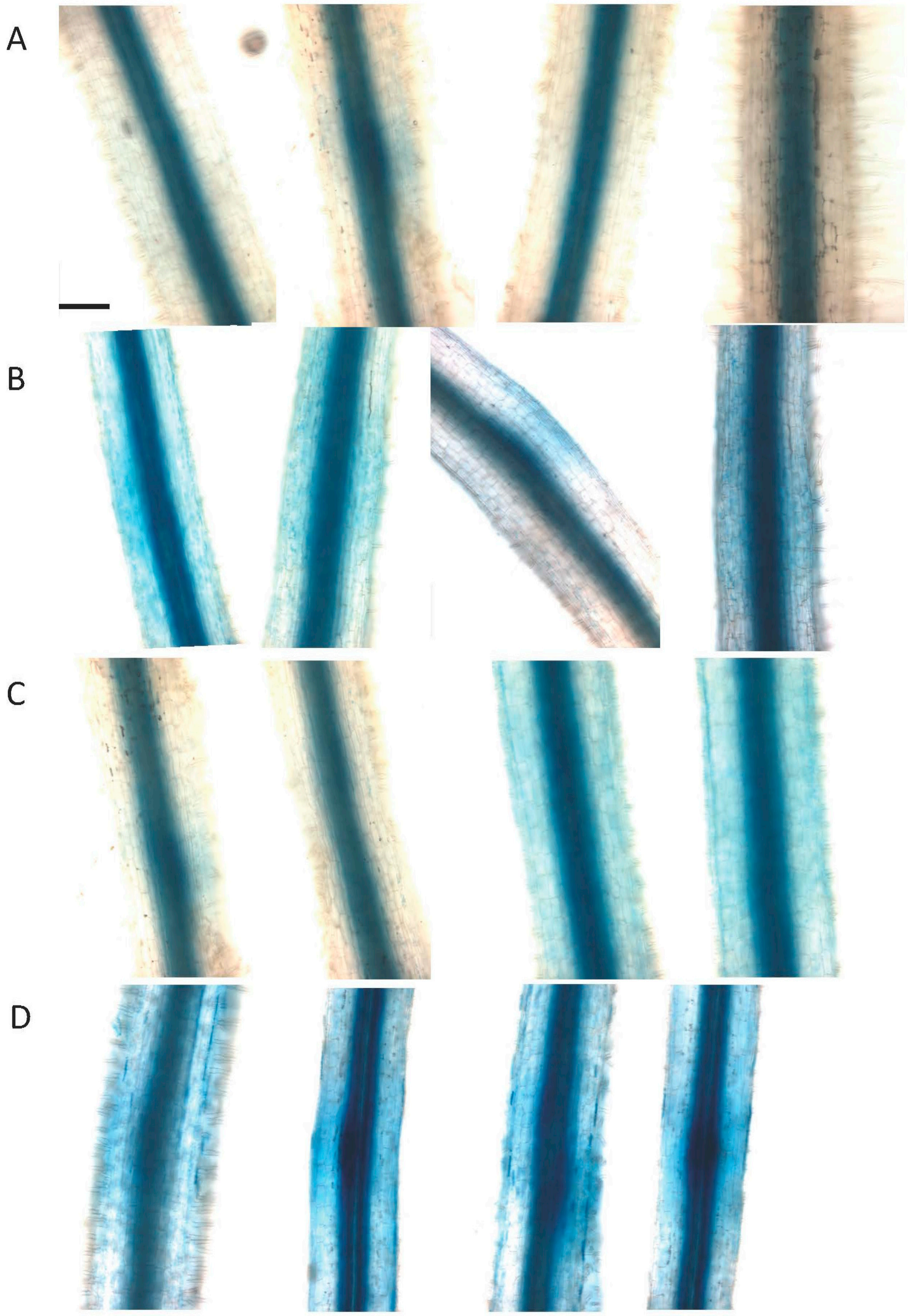

Figure S4. *Cont.*

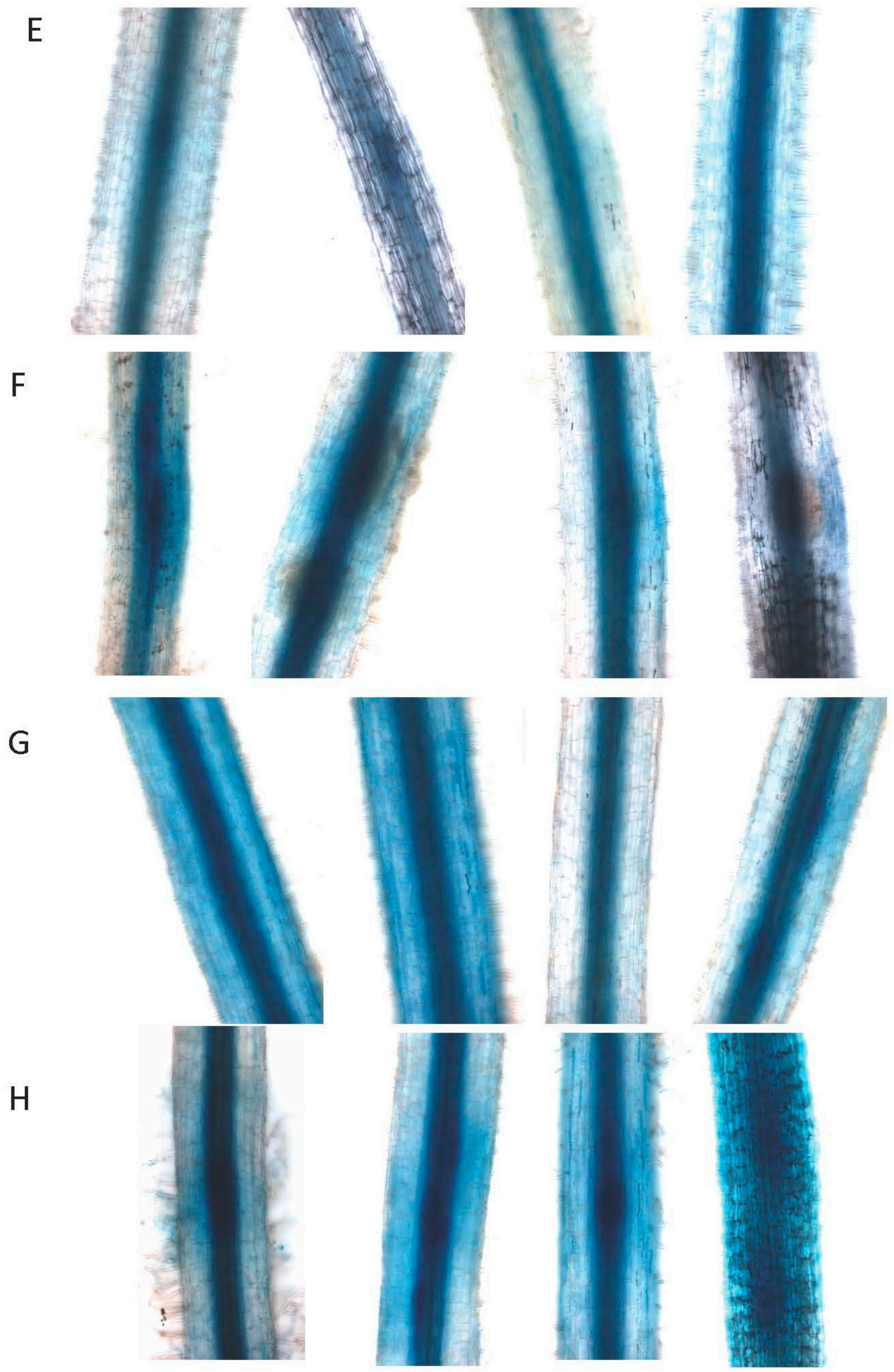

Figure S4. *GH3:GUS* staining in response to nitrate and inoculation. The figure shows representative photos of the inoculation site of roots after GUS staining to indicate the possible variation in staining between individual roots. The inoculation site is located in the middle of each segment shown. (**A**) 24 h p.i. (post inoculation) of no nitrate-treated, uninoculated roots; (**B**) 24 h p.i. of no nitrate-treated, *S. meliloti*-inoculated roots; (**C**) 24 h p.i. of 2.5 mM nitrate-treated, uninoculated roots; (**D**) 24 h p.i. of 2.5 mM nitrate-treated, *S. meliloti*-inoculated roots; (**E**) 48 h p.i. of no nitrate-treated, uninoculated roots; (**F**) 48 h p.i. of no nitrate-treated, *S. meliloti*-inoculated roots; (**G**) 48 h p.i. of 2.5 mM nitrate-treated, uninoculated roots; (**H**) 48 h p.i. of 2.5 mM nitrate-treated, *S. meliloti*-inoculated roots. Magnification bar (in **A**) indicates 500 µm.

Table S1. Summary of protein changes. The table shows the total number of proteins detected and compared across all gels, the number of individual protein spots showing differential display after three way analysis of variance and the number of differentially proteins identified by mass spectrometry. Of the differentially displayed proteins, the number of proteins affected significantly by nitrate (n), inoculation (i), time (t), or interactions of those factors are shown. Many proteins showed significant changes in response to several treatments.

Feature	Number of Proteins
Number of proteins across all gels	1200
Differentially displayed ($p < 0.05$)	248
Identified differentially displayed	106
Proteins affected by nitrate (n)	97
Proteins affected by time (t)	96
Proteins affected by inoculation (i)	38
Proteins affected by n × t	27
Proteins affected by n × i	33
Proteins affected by i × t	18
Proteins affected by n × i × t	22

Table S2. Protein identification by mass spectrometry. The table lists all identified proteins and is subdivided into proteins identified by MALDI-TOF (**A**) and proteins identified by MALDI-TOF/TOF (**B**).

Table S3. Expression patterns of differentially regulated proteins. The table lists all identified, differentially accumulating proteins, and is subdivided into proteins showing a response to inoculation (**A**), an interaction between inoculation and nitrate treatment (**B**), nitrate treatment (**C**), or time point (**D**). Note that some proteins show significant responses to more than one treatment. The graphs showing the expression patterns were scaled to indicate the relative differences in abundance. Absolute abundances of the same proteins are listed in Table S4.

Table S3A. Proteins with a statistically significant ($p < 0.05$) response to nitrate (n), including proteins with a significant interaction between nitrate and time point (nt).

No. [a]	Identification [b]	Function [c]	Gene Id [d]	M [e]	E [f]	Expression Pattern [g]
168	TCP-1/cpn60 chaperonin family protein	Prot. processing	Medtr1g017380	mt	n, nt	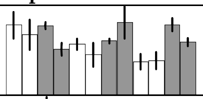
234	Phosphopyruvate hydratase	Prim. Metab	Medtr7g103620	mt	n	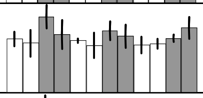
236	F1 ATPase	Energy	Medtr1g108765	mtt	n	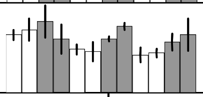
256	N-acyl-L-amino-acid amidohydrolase	Prot. processing	Medtr1g038920	mt	nt	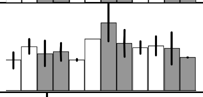
257	F1 ATPase	Energy	Medtr1g108765	mtt	n, t, nt	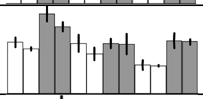
267	Monodehydroascorbate reductase	Defense/stress	Medtr8g098910	mt	n	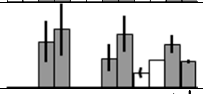
273	Processing peptidase	Prot. processing	Medtr8g102230	mt	n	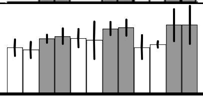

Table S3A. *Cont.*

No. [a]	Identification [b]	Function [c]	Gene Id [d]	M [e]	E [f]	Expression Pattern [g]
280	Tubulin Beta-1 chain	Cell structure	Medtr3g110720	mt	n	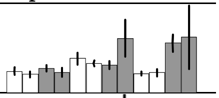
285	2,3-bisphospho-glycerate-independent phosphoglycerate kinase	Prim. Metab	Medtr7g074570	mtt	n	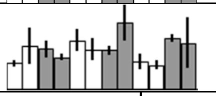
303	S-adenosyl-L-methionine synthetase	Prim. Metab	Medtr2g046710	mt	nt	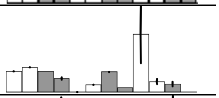
334	NADP-specific glutamate dehydrogenase	Prim. Metab	Medtr6g029460	mt	n	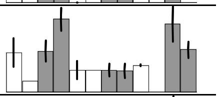
401	ribulose bisphosphate carboxylase/oxygenase activase	Energy	Medtr3g068030	mt	n, t, int	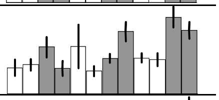
417	Glyceraldehyde-3-phosphate dehydrogenase	Prim. Metab	Medtr3g085850	mtt	t, nt	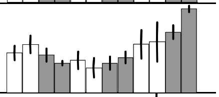
430	Seed linoleate 9S-lipoxygenase	Prim. Metab	Medtr8g018450	mt	n	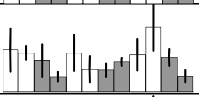
442	TGB12K interacting protein	Unknown	Medtr3g009280	mtt	it, nt, int	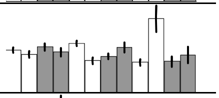
481	O-acetylserine (thiol) lyase	Prim. Metab	Medtr4g087520	mt	n	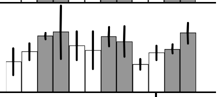
495	pfkB family carbohydrate kinase	Prim. Metab	Medtr2g098950	mt	n	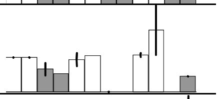
504	pfkB family carbohydrate kinase	Prim. Metab	Medtr2g098950	mtt	n	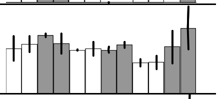
513	pfkB family carbohydrate kinase	Prim. Metab	Medtr2g098950	mt	n, nt	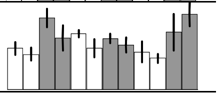
532	Chalcone reductase/Aldo/keto reductase, family oxidoreductase	Sec. Metab	Medtr5g097900	mt	n, nt	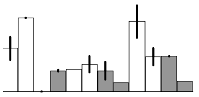
565	Elongation factor 1-beta	Gene regulation	Medtr4g019610	mt	n, t	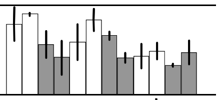
569	Thylakoid-bound ascorbate peroxidase	Defense/ stress	Medtr3g088160	mt	nt	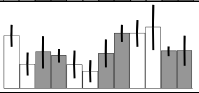
571	Thiosulfate/3-mercaptopyruvate sulfurtransferase	Prim. Metab.	Medtr2g067080	mt	n	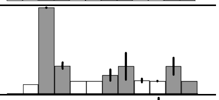
580	Seed specific protein Bn15D17A	Unknown	Medtr6g018270	mt	n	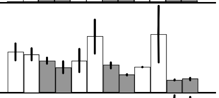
593	S-formylglutathione hydrolase	Prim. Metab	Medtr1g052175	mt	n, t	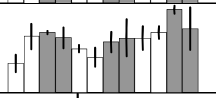
650	26S protease regulatory subunit 6B	Prot. processing	Medtr3g062510	mt	n, t	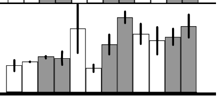

Table S3A. *Cont.*

No. [a]	Identification [b]	Function [c]	Gene Id [d]	M [e]	E [f]	Expression Pattern [g]
659	Metallothionein-like protein type 3	Defense/ stress	Medtr8g060850	mt	n, in	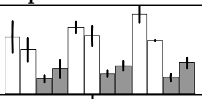
712	Glutathione S-transferase	Defense/ stress	Medtr2g070150	mt	n	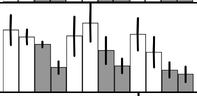
780	Glutathione S-transferase	Defense/ stress	Medtr1g115500	mt	n, t	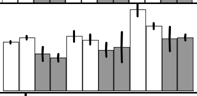
783	Chalcone flavonone isomerase	Sec. metab.	Medtr1g115820	mt	n, in, int	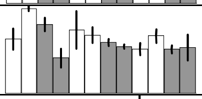
787	NAD(P)H:quinone oxidoreductase, type IV protein	Prim. Metab	Medtr2g011080	mt	n, t	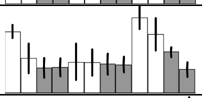
840	Kunitz type trypsin inhibitor	Prot. processing	Medtr3g106585	mtt	n, nt, int	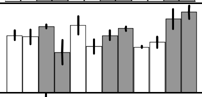
845	Kunitz type trypsin inhibitor / Alpha-fucosidase	Prot. processing	Medtr6g059680	mt	n	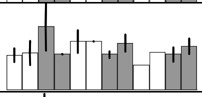
874	Kunitz type trypsin inhibitor / Alpha-fucosidase	Prot. processing	Medtr6g059530	mtt	t, nt, int	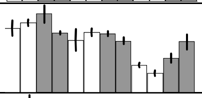
881	ABA-responsive protein	Defense/ stress	Medtr2g035220	mtt	n	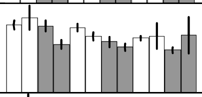
908	ABA-responsive protein	Defense/ stress	Medtr2g035220	mtt	n, t	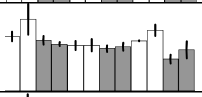
925	Disease-resistance response protein	Defense/ stress	Medtr2g035120	mtt	n, t	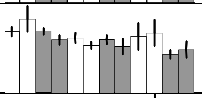
929	ABA-responsive protein	Defense/ stress	Medtr2g035190	mtt	n, nt	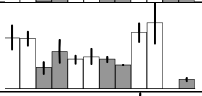
932	Disease-resistance response protein	Defense/ stress	Medtr2g035170	mtt	n, nt	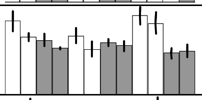
934	ABA-responsive protein	Defense/ stress	Medtr2g035320	mtt	n	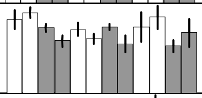
935	ABA-responsive protein	Defense/ stress	Medtr2g035320	mtt	n, nt	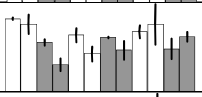
945	Disease-resistance response protein	Defense/ stress	Medtr2g035150	mtt	n, t, nt	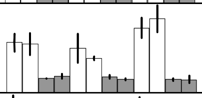
946	ABA-responsive protein	Defense/ stress	Medtr2g035320	mtt	n	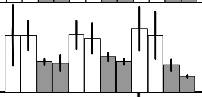
976	ABA-responsive protein	Defense/ stress	Medtr2g035320	mtt	n	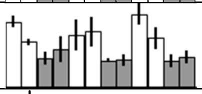
1011	RNA-binding (RRM/RBD/RNP motif) family protein	Gene regulation	Medtr3g084040	mtt	n	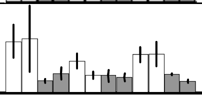

Table S3A. *Cont.*

No. [a]	Identification [b]	Function [c]	Gene Id [d]	M [e]	E [f]	Expression Pattern [g]
1020	Elongation factor 1-beta	Gene regulation	Medtr4g019610	mt	n, t	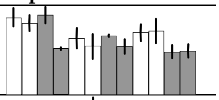
1026	Disease-resistance response protein	Defense/ stress	Medtr2g035105	mtt	n	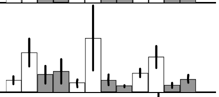
1038	Phosphopyruvate hydratase	Prim. Metab.	Medtr7g103620	mtt	n	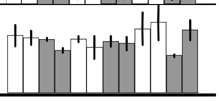

Table S3B. Proteins with a statistically significant ($p < 0.05$) response to inoculation (i), including proteins with a significant interaction between inoculation and time point (it).

No. [a]	Identification [b]	Function [c]	Gene Id [d]	M [e]	E [f]	Expression Pattern [g]
65	Protein disulfide isomerase-like protein	Prot. processing	Medtr2g094180	mtt	it	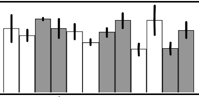
156	Protein disulfide-isomerase precursor	Prot. processing	Medtr3g088220	mtt	i, t	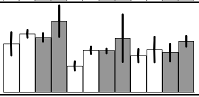
306	Limonoid UDP-glucosyltransferase-like protein/(Iso)flavonoid glycosyltransferase	Sec. Metab	Medtr1g107285	mt	i	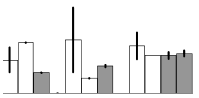
336	NADP-specific glutamate dehydrogenase	Prim. Metab	Medtr7g085630	mtt	it	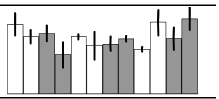
361	Actin	Cell structure	Medtr7g026230	mt	i	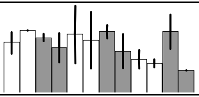
442	TGB12K interacting protein	Unknown	Medtr3g009280	mtt	it, nt, int	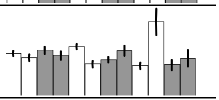
456	Zinc-binding dehydrogenase family oxidoreductase	Energy	Medtr7g034395	mt	i, int	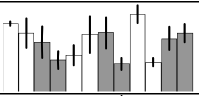
457	Plasma membrane-associated-like protein	Transport	Medtr2g042430	mt	i	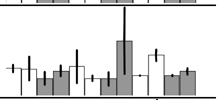
491	Transducin/WD40 repeat protein	Signalling	Medtr4g105700	mt	i	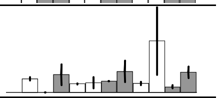
541	Isoflavone reductase/Phenylcoumaran benzylic ether reductase-like protein Fi1	Sec. Metab	Medtr5g020740	mt	i, t	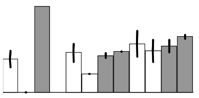
699	20S proteasome subunit alpha	Prot. processing	Medtr1g112250	mtt	i	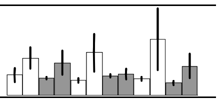
741	RNA-binding (RRM/RBD/RNP motif) family protein	Gene regulation	Medtr7g013730	mtt	i	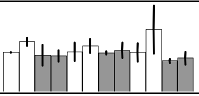
875	Pathogenesis-related protein bet V I family protein	Defense/ stress	Medtr8g045665	mt	i	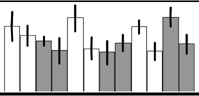

Table S3B. *Cont.*

No. [a]	Identification [b]	Function [c]	Gene Id [d]	M [e]	E [f]	Expression Pattern [g]
890	Disease-resistance response protein	Defense/ stress	Medtr2g035120	mtt	it	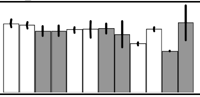
901	Pathogenesis-related protein bet V I family protein	Defense/ stress	Medtr8g045640	mt	it	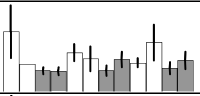
1040	Profilin-1,	Cell structure	Medtr8g102330	mtt	it	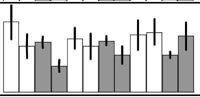
1142	Acid invertase	Prim. Metab	Medtr4g101630	mt	i, t	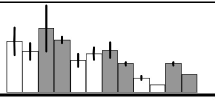

Table S3C. Proteins with a statistically significant ($p < 0.05$) interaction between inoculation and nitrate (in), including proteins with a significant interaction between inoculation, nitrate and time point (int).

No. [a]	Identification [b]	Function [c]	Gene Id [d]	M[e]	E [f]	Expression pattern [g]
35	RNA-binding KH domain protein	Gene regulation	TC95861	mtt	in	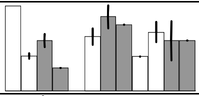
238	UTP-glucose-1-phosphate uridylyltransferase	Prim. Metab	Medtr5g077000	mtt	t, int	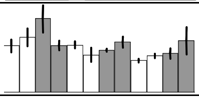
249	Tubulin beta-1 chain	Cell structure	Medtr4g019110	mt	t, int	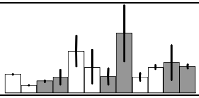
355	Fructose-bisphosphate aldolase	Prim. Metab	Medtr5g069055	mtt	int	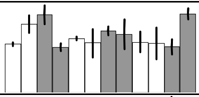
401	Ribulose bisphosphate carboxylase/oxygenase activase	Energy	Medtr3g068030	mt	n, t, int	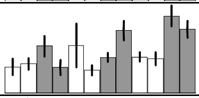
414	Pyruvate dehydrogenase E1 component beta subunit	Prim. Metab	Medtr7g005380	mtt	int	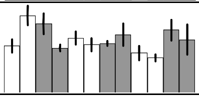
442	TGB12K interacting protein	Unknown	Medtr3g009280	mtt	it, nt, int	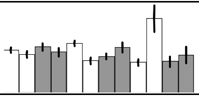
456	Zinc-binding dehydrogenase family oxidoreductase	Energy	Medtr7g034395	mt	i, int	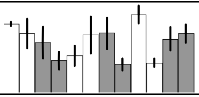
488	Branched-chain-amino-acid aminotransferase	Prim. Metab	Medtr5g014960	mt	in	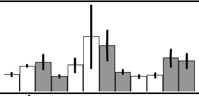
515	Isoflavone reductase-like protein Bet protein	Defense/ stress	Medtr3g111980	mt	in	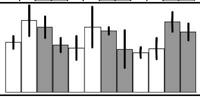
567	20S proteasome subunit alpha type 1	Prot. processing	Medtr1g022410	mt	t, int	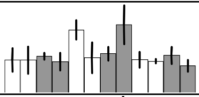
596	Papain family cysteine protease	Prot. processing	Medtr1g018840	mt	t, int	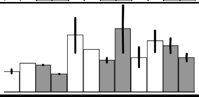

Table S3C. *Cont.*

No. [a]	Identification [b]	Function [c]	Gene Id [d]	M [e]	E [f]	Expression Pattern [g]
641	Glutathione S-transferase	Defense/ stress	Medtr1g115500	mt	in	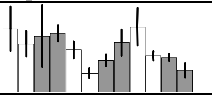
658	Caffeoyl-CoA *O*-methyltransferase	Sec. Metab	Medtr4g085590	mt	t, in	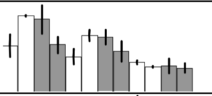
659	Metallothionein-like protein type 3	Defense/ stress	Medtr8g060850	mt	n, in	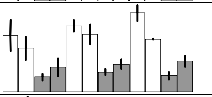
716	Triosephosphate isomerase	Prim. Metab.	Medtr2g096940	mt	in	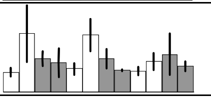
783	Chalcone-flavanone isomerase family protein	Sec. Metab.	Medtr1g115820	mt	n, in, int	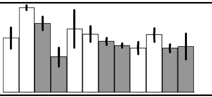
840	Kunitz type trypsin inhibitor	Prot. processing	Medtr3g106585	mtt	n, nt, int	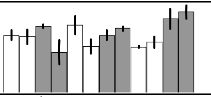
874	Kunitz type trypsin inhibitor/ Alpha-fucosidase	Prot. processing	Medtr6g059530	mtt	t, nt, int	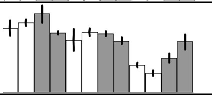
898	Pathogenesis-related protein bet V I family protein	Defense/ stress	Medtr8g045490	mt	in	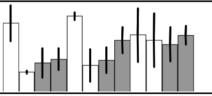
922	Disease-resistance response protein	Defense/ stress	Medtr2g035120	mtt	t, int	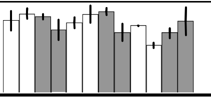

Table S3D. Proteins with a statistically significant ($p < 0.05$) response to the time point (t).

No. [a]	Identification [b]	Function [c]	Gene Id [d]	M [e]	E [f]	Expression Pattern [g]
15	Heat shock protein 70	Prot. processing	Medtr7g099680	mt	t	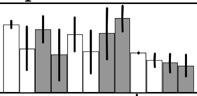
37	RNA-binding KH domain protein	Gene regulation	Medtr1g016760	mtt	t	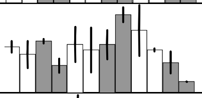
52	Heat shock protein 81-2	Prot. processing	Medtr1g016760	mt	t	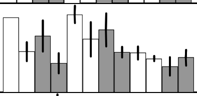
61	BHLH transcription factor	Gene regulation	Medtr7g053410	mt	t	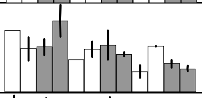
114	NLI interacting factor-like phosphatase	Transport	TC109645	mt	t	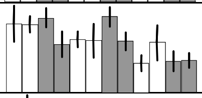
123	Myosin heavy chain	Cell structure	Medtr1g015620	mt	t	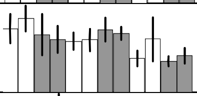
147	Protein disulfide isomerase	Prot. processing	Medtr3g088220	mtt	t	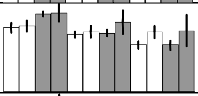
156	Protein disulfide isomerase	Prot. processing	Medtr3g088220	mtt	i, t	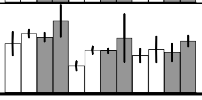

Table S3D. *Cont.*

No. [a]	Identification [b]	Function [c]	Gene Id [d]	M [e]	E [f]	Expression Pattern [g]
192	Gutathione reductase	Defense/stress	Medtr6g033515	mt	t	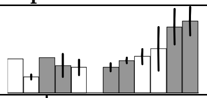
238	UTP-glucose-1-phosphate uridylyltransferase	Prim. Metab	Medtr5g077000	mtt	t, int	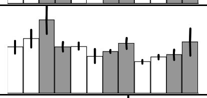
249	Tubulin beta-1 chain	Cell structure	Medtr4g019110	mt	t, int	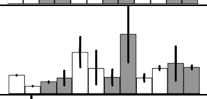
250	Phosphopyruvate hydratase	Prim. Metab	Medtr7g103620	mt	t	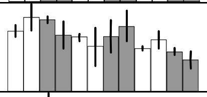
257	F1 ATPase	Energy	Medtr1g108765	mtt	n, t, nt	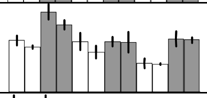
309	S-adenosyl-L-methionine synthetase	Prim. Metab	Medtr7g110310	mt	t	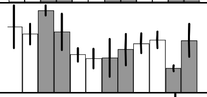
401	Ribulose bisphosphate carboxylase/oxygenase activase	Energy	Medtr3g068030	mt	n, t, int	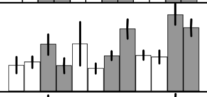
408	Metallothionein-like protein type 3	Defense/stress	Medtr8g060850	mtt	t	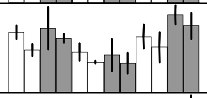
417	Metallothionein-like protein type 3	Defense/stress	Medtr8g060850	mtt	t, nt	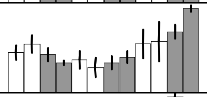
427	Fructose-bisphosphate aldolase	Prim. Metab	Medtr5g069055	mtt	t	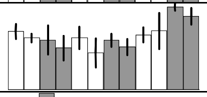
541	Isoflavone reductase	Sec. Metab	TC100786	mt	i, t	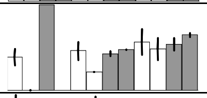
565	Translational elongation factor 1	Gene regulation	TC106814	mt	n, t	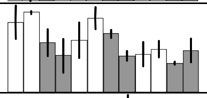
567	20S proteasome subunit PAF2	Prot. processing	TC107025	mt	t, int	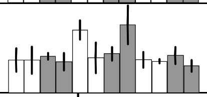
586	Phenylcoumaran benzylic ether reductase-like protein Fi1	Prim. Metab	Medtr5g020740	mt	t	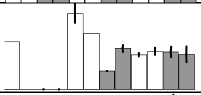
593	S-formylglutathione hydrolase	Prim. Metab	Medtr1g052175.1	mt	n, t	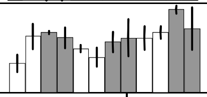
596	Papain family cysteine protease	Prot. processing	Medtr1g018840	mt	t, int	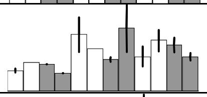
608	Cyclin	Cell structure	Medtr7g089080	mt	t	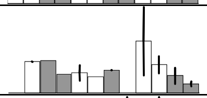
615	Caffeoyl-CoA O-methyltransferase	Sec. Metab	Medtr4g085590	mtt	t	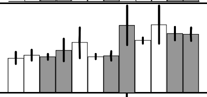
633	Hydroxyacylglutathione hydrolase	Prim. Metab	Medtr5g068440	mt	t	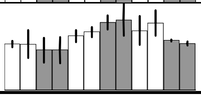

Table S3D. *Cont.*

No. [a]	Identification [b]	Function [c]	Gene Id [d]	M [e]	E [f]	Expression Pattern [g]
650	26S protease regulatory subunit 6B	Prot. processing	Medtr3g062510	mt	n, t	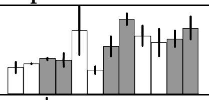
658	Caffeoyl-CoA 3-O-methyltransferase	Sec. Metab	Medtr4g085590	mt	t, in	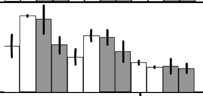
780	Glutathione S-transferase	Defense/stress	Medtr1g115500	mt	n, t	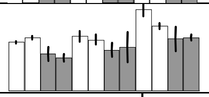
787	NAD(P)H:quinone oxidoreductase, type IV protein	Prim. Metab	Medtr2g011080	mt	n, t	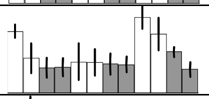
819	ATP synthase D chain	Energy	Medtr1g095830	mt	t	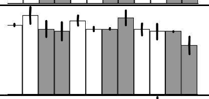
820	Oxygen-evolving enhancer protein 2-1	Energy	Medtr3g449930	mt	t	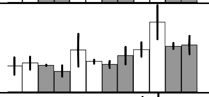
843	Type II peroxiredoxin	Defense/stress	Medtr2g022660	mt	t	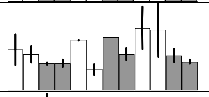
874	Kunitz type trypsin inhibitor / Alpha-fucosidase	Prot. processing	Medtr6g059530	mtt	t, nt, int	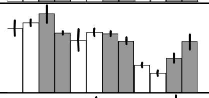
896	Polyketide cyclase/dehydrase and lipid transporter	Defense/stress	Medtr8g045300	mt	t	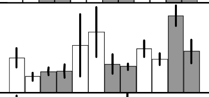
905	Disease-resistance response protein	Defense/stress	Medtr2g035120	mtt	t	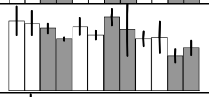
908	ABA-responsive protein	Defense/stress	Medtr2g035220	mtt	n, t	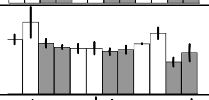
922	Disease-resistance response protein	Defense/stress	Medtr2g035120	mtt	t, int	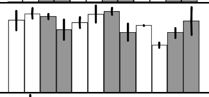
925	Disease-resistance response protein	Defense/stress	Medtr2g035120	mtt	n, t	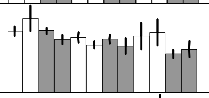
945	Disease-resistance response protein	Defense/stress	Medtr2g035150	mtt	n, t, nt	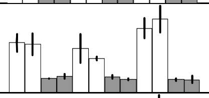
951	STIG1	Unknown	Medtr4g044463	mt	t	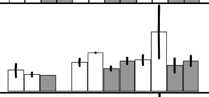
958	RNA-binding (RRM/RBD/RNP motif) family protein	Gene regulation	Medtr4g070080	mtt	t	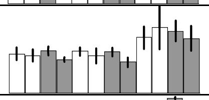
970	Ubiquitin-conjugating enzyme	Prot. processing	Medtr2g078010	mtt	t	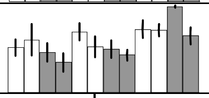
978	Polyketide cyclase/dehydrase and lipid transporter	Defense/stress	Medtr1g030820	mt	t	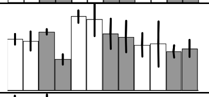
1020	Elongation factor 1-beta	Gene regulation	Medtr4g019610	mt	n, t	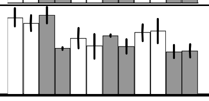

Table S3D. *Cont.*

No. [a]	Identification [b]	Function [c]	Gene Id [d]	M [e]	E [f]	Expression Pattern [g]
1057	Type II peroxiredoxin	Defense/ stress	Medtr6g087990	mt	t	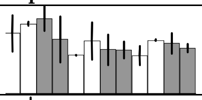
1076	2,3-bisphosphoglycerate-independent phosphoglycerate mutase	Prim. Metab	Medtr7g074570	mt	t	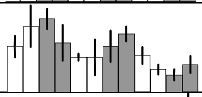
1129	Glutathione-S-transferase	Defense/ stress	Medtr1g067180	mt	t	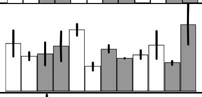
1142	Acid invertase	Prim. Metab	Medtr4g101630	mt	i,t	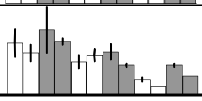

[a] 'No' refers to the identification number used throughout this study, and corresponds to the numbers shown in Supplement Figure 3; [b] 'Identification' shows the annotation of the in the *M. truncatula* genome 4.0v1 index; [c] 'Function' indicated the likely functional classification of the annotated protein in the cell, according to Bevan et al. (1998). Prim. Metab. = primary metabolism; Sec. Metab. = secondary metabolism; Prot. Processing = protein processing; [d] 'Gene ID' refers to the Locus Id in the *M. truncatula* genome 4.0v1 index; [e] 'M' indicated the method of identification by mass spectrometry as either MALDI-TOF (mt) or MALDI-TOF/TOF (mtt); [f] 'E' indicates the statistically significant effects from either the nitrate treatment (n), inoculation with *S. meliloti* (i), time point (t), or interactions of these treatments. The effects were established from three way analyses of variance using the restricted maximum likely hood method (REML); [g] 'Expression pattern' shows the means of protein abundance as % volume of each protein on a gel. Each bar represents the mean of abundance of the three biological repeats, error bars indicate standard error. White bars are from treatments without nitrate, grey bars are from treatments with 2.5 mM nitrate. From left to right the bars represent the following treatments: 24 h control no nitrate, 24 h inoculated no nitrate, 24 h control 2.5 mM nitrate, 24 h inoculated 2.5 mM nitrate, 48 h control no nitrate, 48 h inoculated no nitrate, 48 h control 2.5 mM nitrate, 48 h inoculated 2.5 mM nitrate, 5 days control no nitrate, 5 days inoculated no nitrate, 5 days control 2.5 mM nitrate, 5 days inoculated 2.5 mM nitrate.

Table S4. Relative protein abundance of the 106 identified proteins
